# Cardiac electrophysiological responses to traffic pollution in adults with or without chronic cardiopulmonary diseases

**DOI:** 10.1016/j.envint.2025.109764

**Published:** 2025-09

**Authors:** Xin Meng, Yan Lin, Jicheng Gong, Peter Collins, Sabine Ernst, Wu Chen, Meilin Yan, Junfeng Jim Zhang, Kian Fan Chung

**Affiliations:** a SKL-ESPC & SEPKL-AERM, College of Environmental Sciences and Engineering, and Center for Environment and Health, Peking University, Beijing, China; b Nicholas School of the Environment & Duke Global Health Institute, Duke University, Durham, NC, USA; c National Heart & Lung Institute, Imperial College London & Royal Brompton & Harefield Hospital, London, UK; d Department of Population and Public Health Sciences, Keck School of Medicine, University of Southern California, Los Angeles, CA, USA; e Department of Environmental Science and Engineering, School of Light Industry Science and Engineering, Beijing Technology and Business University, Beijing, China

**Keywords:** Cardiac effects, Traffic pollution, Susceptibility, Chronic obstructive pulmonary disease, Nitro linoleic acids

## Abstract

**Background:**

Traffic pollution exposure has been associated with adverse cardiovascular outcomes, but determinants of individual susceptibility remain unclear. To explore whether disease status modifies traffic-related cardiac responses and to examine the cardioprotective role of nitro linoleic acids.

**Methods:**

In a crossover study, 39 chronic obstructive pulmonary disease (COPD) patients, 38 ischemic heart disease (IHD) patients, and 39 healthy participants walked for two hours on traffic-congested Oxford Street and in traffic-free Hyde Park, in random order, on separate days. Cardiac electrical activity, including heart rate, heart rate variability (HRV), QT interval, and ST-segment changes, was continuously monitored for 24 h. At 24 h following the walk, a urine void was collected and analyzed for nitro linoleic acid NO_2_-cLA (a cardioprotective marker). Mixed-effect models assessed pollution-related cardiac changes.

**Results:**

In reference to walking in the park, participants following the street walk exhibited adverse cardiac changes, including increased heart rate, decreased HRV, shortened QT interval, and elevated ST-segment, with larger changes in some parameters observed in COPD participants, though between-group differences were not statistically significant. Among COPD participants, the cardiac effects were more pronounced in those who did not use inhaler medications than in those who used them. NO_2_-cLA concentrations were significantly lower in COPD participants after the street walk compared to after the park walk (0.0189 vs 0.0323 μg/g creatinine, p = 0.03). Increasing NO_2_-cLA concentrations were associated with increased pNN50 and QTc and decreased ST elevation. Among measured pollutants, ultrafine particles and black carbon were most elevated in the street and showed the strongest cardiac effects.

**Conclusions:**

Although between-group comparisons were not statistically significant, COPD patients showed numerically larger electrophysiological changes and reductions in NO_2_-cLA levels following short-term exposure to traffic pollution. Use of inhaler medications lessened these effects, suggesting a potential role of medication use in modifying pollution responses.

## Highlights

• COPD participants exhibited greater electrophysiological responses to traffic pollution compared to those with IHD and healthy individuals.
• In COPD participants, NO_2_-FA levels increased compensatorily but declined most markedly after traffic pollution exposure.
• Inhaler use in COPD participants was associated with attenuated electrocardiographic changes following traffic pollution exposure.

## 1 Introduction

Traffic-related air pollution is a significant contributor to global cardiovascular diseases (Roth et al., 2020, Pant and Harrison, 2013). Diesel exhaust particles, categorized as a Group 1 carcinogen (Iarc, 2014), are associated with increased mortality from circulatory and ischemic heart diseases (Boogaard et al., 2022). However, further research is needed to identify vulnerable populations affected by these cardiac effects, emphasizing the importance of targeted studies to protect at-risk groups.

Individuals with cardiopulmonary conditions, like chronic obstructive pulmonary disease (COPD) and ischemic heart disease (IHD), may exhibit susceptibility to traffic-related health threats compared to healthy individuals. We used a quasi-experimental design to replicate real-world exposure scenarios, involving individuals with COPD or IHD walking along a roadside of a busy city street in London, UK, in comparison to walking in a traffic-free urban park. Previously, we reported that a 2-h traffic exposure during walking induced acute and sustained adverse respiratory and cardiovascular effects, including bronchoconstriction and increased arterial stiffness (McCreanor et al., 2007, Sinharay et al., 2018). We have yet to examine the potential effects of this natural traffic pollution exposure on cardiac function measured as autonomic tone indicators.

Previous studies have shown that traffic-related pollution contributes to cardiovascular morbidity and mortality. One of the pathophysiologic mechanisms is that particulate matter, a principal component of traffic air pollution mixture, can disrupt autonomic tone, including reducing heart rate variability (HRV), delaying cardiac conductance, and increasing ischemia as evidenced by ST-segment elevation on the electrocardiogram (Miller and Newby, 2020, Brook et al., 2010, Weichenthal et al., 2014, Yang et al., 2018). However, susceptibility by disease status and factors (e.g., regular medication use) that may affect susceptibility remains poorly understood with regard to cardiac responses to traffic air pollution. Individuals with underlying cardiopulmonary conditions could face higher health risks from traffic pollution exposure. Though these populations often use medications such as inhalers (containing β-adrenergic agonists, anticholinergics, and corticosteroids) and β-blockers to manage their conditions, it remains unclear whether these treatments effectively mitigate the cardiac risks associated with traffic pollution.

Individual susceptibility to traffic pollution may also be influenced by key signaling molecules involved in cardiovascular mechanisms. Recent studies have shown the cardioprotective properties of nitrated polyunsaturated fatty acids (e.g., NO_2_-cLA), which can prevent ischemic ventricular arrhythmias (Villacorta et al., 2016, Mollenhauer et al., 2018). NO_2_-cLA is an endogenous protective molecule synthesized during inflammatory or ischemic events. Given its biological function, we hypothesize that NO_2_-cLA may modulate the cardiovascular effects of traffic pollution exposure, especially in individuals with pre-existing cardiopulmonary conditions.

In this randomized crossover study, middle-aged and elderly individuals with COPD and IHD, as well as healthy participants, were recruited to walk alongside a busy vehicle-traffic-polluted street and, on another occasion, in a large public park with green space. This study builds upon a previous investigation of the same participant cohort, which primarily focused on respiratory outcomes and arterial stiffness (Sinharay et al., 2018). The current analysis uniquely examines cardiac electrophysiological responses and the role of urinary NO_2_-cLA as a potential marker of pollution-related cardiovascular effects. We hypothesized that traffic-related pollution may trigger acute changes in cardiac electrical activities and increase the risk of arrhythmic events, particularly in individuals with chronic cardiopulmonary diseases. This study aimed to examine disease status and medication use as susceptible factors and to explore whether NO_2_-cLA level is related to susceptibility to traffic-induced cardiac effects.

## 2 Methods

### 2.1 Study design and participants

The study was conducted between 2012 and 2014 and shares the same participant cohort as Sinharay et al., and the detailed study design has been described previously (Sinharay et al., 2018). Briefly, the study was a randomized crossover study to assess the cardiorespiratory health effects of short-term exposure to traffic air pollution. With a 3- to 8-week washout period, each participant underwent a 2-h walk in either a traffic-heavy scenario (i.e., Oxford Street, London) or a traffic-free green space (i.e., Hyde Park), with order randomized across participants. At the time of the study, only buses and official taxi cabs were allowed to travel through Oxford Street, and all buses and taxi cabs were powered with diesel engines. From existing databases or outpatient respiratory and cardiology clinics at the Royal Brompton & Harefield Hospital, 119 current non-smokers, consisting of 40 COPD patients, 39 IHD patients, and 40 age-matched individuals without COPD and IHD (regarded as the healthy control), were recruited. COPD was diagnosed based on post-bronchodilator spirometry according to GOLD criteria: FEV_1_/FVC < 0.70 and FEV_1_ ≤ 80 % predicted. IHD was confirmed by angiographic evidence of coronary artery disease from medical records. Cardiorespiratory measurements before, during, and after the 2-h walk were made at time points up to 24 h following the walk. The electrocardiogram (ECG) of these participants during their walks was continuously recorded using 24-h Holter monitors. Prescribed medications were routinely taken as recommended by their physicians and classified into inhaler therapy consisting of β-agonists, anticholinergics, and corticosteroids, oral β-blockers, and other cardiac medicines. The study was approved by the UK National Research Ethics Service (London City Road and Hampstead Ethics Committee; Research Ethics Number 12/LO/1064). Each participant gave informed signed consent to participate.

### 2.2 Exposure measurement

We monitored ambient air pollution and noise during each 2-h walk session on Oxford Street and in Hyde Park, respectively. Particulate air pollutants included ultrafine particle number concentrations (PNC; size range of 10–300 nm) measured via NanoTracer (Philips Aerosense), black carbon measured via AE51 (microAeth Black Carbon aerosol monitor, AEthlabs, CA, USA), and particles with an aerodynamic diameter of 2.5 μm or less (PM_2.5_) and 10 μm or less (PM_10_) measured via AM510 (SidePak Personal Aerosol Monitors, TSI Ltd, MI, USA). NO_2_ is a gaseous pollutant emitted by vehicles and its concentrations were obtained from the two nearby stationary monitoring sites on Oxford Street and in Hyde Park. Noise levels were measured by a noise meter (Bruel and Kjaer Type 2236 Sound level meter, Naerum, Denmark). Temperature and relative humidity were simultaneously logged.

### 2.3 Cardiac outcome measurement

During each session, participants received a 12-lead Holter (H12+, Mortara, USA) 1 h before the intervention and wore it for up to 24 h. Quantitative indexes were calculated for each hour including heart rate and time-domain HRV metrics regarding the proportion of the number of pairs of adjacent normal-to-normal (NN), intervals differing by more than 50 ms of the entire recording (pNN50), square root of the mean of the sum of the squares of differences between adjacent NN intervals (rmsSD), the standard deviation of the averages of NN intervals in all 5 min segments (SDANN), the standard deviation of all NN intervals (SDNN), and the total number of all NN intervals divided by the height of the histogram of all NN with bins of 7.8125 ms (Tri); the duration of QT interval, corrected for heart rate (QTc) using Bazett's formula; b maximum elevation and maximum depression of ST-segment changes across all 12 ECG leads which reflect the most extreme ST-segment shifts rather than derived from a single fixed lead. The onset of arrhythmia was a binary outcome as to whether the electrocardiographic recordings indicated the occurrence of ventricular ectopy or supraventricular ectopy during each session. All ECG data were obtained using the Mortara H12+ Holter system and processed according to the manufacturer's standard procedures. One cardiologist (Dr. Ernst Sabine) visually inspected every recording to ensure data completeness and the absence of major artifacts. Electrical noise interference that led to a non-interpretable ECG signal was noted and these time intervals were removed from the analysis.

### 2.4 NO_2_-cLA measurement

We used an HPLC-MS/MS system (TSQ Quantum Access Max, Thermo Fisher Scientific, MA, USA) to measure urinary levels of two nitrated conjugated linoleic acids (NO_2_-cLA), namely 9(E),11(E)-9-NO_2_-cLA and 9(E),11(E)-12-NO_2_-cLA. The pretreatment of urine samples consists of enzymatic deconjugation with β-glucuronidase-arylsulfatase and solid-phase extraction using a Bond Elute Certify II cartridge, with a detailed procedure introduced previously (Lin et al., 2024). We spiked deuterated 10-nitrooleic acid into each sample before the pretreatment as the internal standard. A Kinetex 2.3 µm C18 column (100 × 2.1 mm, Phenomenex, CA, USA) was used for chromatographic separation. The ion pairs of 324/277 *m*/*z* for NO_2_-cLA and 343/296 *m*/*z* for deuterated 10-nitrooleic acid were used for quantification. The instrumental limit of detection (LOD) was 0.02 ng/ml. We detected NO_2_-cLA in 76.3 % of the urine samples. The average (standard deviation) method recovery for NO_2_-cLA is 93.7 (16.5) %. The urinary NO_2_-cLA concentrations were adjusted by urinary creatinine concentrations measured based on the Jaffe reaction (Toora and Rajagopal, 2002).

### 2.5 Statistical analysis

We calculated demographic and cardiac outcome statistics at baseline for each subgroup by disease status (healthy, IHD, and COPD) and walking sites (Oxford Street and Hyde Park). For the main hypothesis, we tested whether short-term exposure to the traffic-related scenario worsened cardiac responses using linear mixed-effects models. Dependent variables were the 24-h average ECG indicator changes from baseline values. The models factored in an interaction of site and disease status as the fixed effect and participant as a random effect to account for the within-person correlation of health endpoints. We also controlled for ambient temperature and relative humidity. Several covariant correlation structures were tested, including autoregressive processes, symmetry structure, and spatial correlations, and the best models were determined by the Akaike information criterion. Regarding the binary outcome of arrhythmia, we applied logistic regression models to assess the effects of walking in the two sites on the onset of any arrhythmia. More specifically, to explore when the pollution effects emerged and whether they differed by disease group, we applied mixed-effects models with a three-way interaction term (site × group × time) as fixed effects. The dependent variables were hourly ECG values relative to baseline, and time was treated as a categorical variable (0–23 h). Models included random intercepts for participants and were adjusted for ambient conditions. Furthermore, to explore the specified role of traffic-related air pollution and noise, we assessed health outcome changes associated with an interquartile range increase in PNC, black carbon, PM_2.5_, PM_10_, NO_2_, and noise, using mixed-effects models but substituting site with pollutant concentration. Furthermore, we conducted post hoc stratified analyses to investigate the potential modification of medication use on the cardiac effects of traffic pollution. We also examined the role of NO_2_-cLA in association with ECG changes using mixed-effects models. Statistical analyses were performed using the nlme and lmeTest packages of R software (version 3.2.3, R Development Core Team).

## 3 Results

### 3.1 Participants and traffic exposure levels

Among the 119 participants, the vast majority (n = 116) had complete ECG recordings. The subject characteristics are shown in Table 1. The three subgroups were age-matched, with a median age of 66.0 years. Of the IHD participants, 87.2 % were male while the sex composition was balanced among healthy and COPD participants. The body mass index of participants with IHD (median: 26.5 kg/m^2^) and COPD (26.0 kg/m^2^) was higher than the healthy group (22.4 kg/m^2^). All COPD participants were non-smokers at the time of recruitment, and all but one (94.9 %) were previous smokers. According to self-reported data, 28 of 38 (73.3 %) COPD participants used their inhalers; 13 of 39 (33.3 %) IHD participants were on β-blockers; and 15 of 39 (38.5 %) IHD patients were on other cardiac medicines, including antihypertensive agents, antianginal medications, and statins. The baseline health indicator levels are summarized by disease status and exposure site in Table 1 and Supplemental Table S1, respectively.

**Table 1:** Baseline characteristics of participants.

	Healthy	IHD	COPD	Overall
	(N = 39)	(N = 39)	(N = 38)	(N = 116)
Age, year	62.0 [8.5]	68.0 [11.5]	69.0 [9.3]	66.0 [11.0]
Sex				
Female	21 (55.3 %)	5 (12.8 %)	21 (53.8 %)	47 (40.5 %)
Male	17 (44.7 %)	34 (87.2 %)	18 (46.2 %)	69 (59.5 %)
BMI, kg/m^2^	22.4 [6.7]	26.5 [4.6]	26.0 [6.6]	25.8 [6.3]
Smoking				
Never smoker	26 (68.4 %)	16 (41.0 %)	1 (2.6 %)	43 (37.1 %)
Past smoker	12 (31.6 %)	23 (59.0 %)	37 (94.9 %)	72 (62.1 %)
Smoking history, pack-years	0.0 [0.1]	3.5 [13.4]	35.0 [32.8]	4.1 [30.0]
Medication				
Inhaler^a^	0	0	28 (73.7 %)	28 (24.1 %)
β-blocker^b^	1 (2.6 %)	13 (33.3 %)	1 (2.6 %)	15 (12.9 %)
Other cardiac medicine^c^	2 (5.1 %)	15 (38.5 %)	4 (10.5 %)	21 (18.1 %)
Any other drugs	14 (35.9 %)	8 (20.5 %)	1 (2.6 %)	23 (19.8 %)
None medication	22 (56.4 %)	3 (7.7 %)	4 (10.5 %)	29 (25.0 %)
Heart rate, bpm	69.5 [10.0]	64.0 [12.0]	76.0 [15.0]	70.0 [15.0]
pNN50, %	6.00 [14.0]	3.50 [7.0]	3.00 [8.5]	4.00 [10.0]
rmsSD, ms	30.0 [20.0]	26.0 [13.5]	25.0 [19.0]	28.0 [19.0]
SDNN, ms	73.0 [33.5]	70.0 [21.0]	57.0 [31.0]	66.0 [30.0]
SDANN, ms	64.0 [23.5]	64.0 [25.0]	56.0 [26.5]	61.0 [28.3]
Tri, ms	24.0 [7.0]	21.5 [8.75]	18.0 [6.5]	21.0 [9.8]
QT, ms	391 [32.0]	415 [31.0]	386 [36.0]	398 [39.0]
QTc, ms	411 [16.0]	418 [25.0]	413 [18.5]	414 [20.0]
ST depression, μV	−44.0 [−33.0]	−46.0 [−41.0]	−37.0 [−26.0]	−42.0 [−31.0]
ST elevation, μV	113 [62.0]	117 [89.0]	104 [64.0]	113 [75.0]
NO_2_-cLA, μg/g creatinine^d^	0.0123 [0.0478]	0.0111 [0.0703]	0.0228 [0.195]	0.0174 [0.0696]

Data are median [IQR] or N (%). Bpm: beats per minute; COPD: Chronic obstructive pulmonary disease; IHD: Ischaemic heart Disease; ms: milliseconds; μV: microvolts.

a β-adrenergic agonist, anticholinergics, and inhaled corticosteroid.

b β-blocker included Atenolol, Bisoprolol, and Metoprolol.

c Other cardiac medicine included Doxazosin, Enalapril, Lisinopril, Ramipril, Amlodipine, Adalat, Tildiem, Isosorbide Mononitrate, Nicorandil, Isosobibe, Irbesartan, Losartan, and Perindopril Erbumine.

d The concentration is corrected by urinary creatinine.

**Fig. 1:**
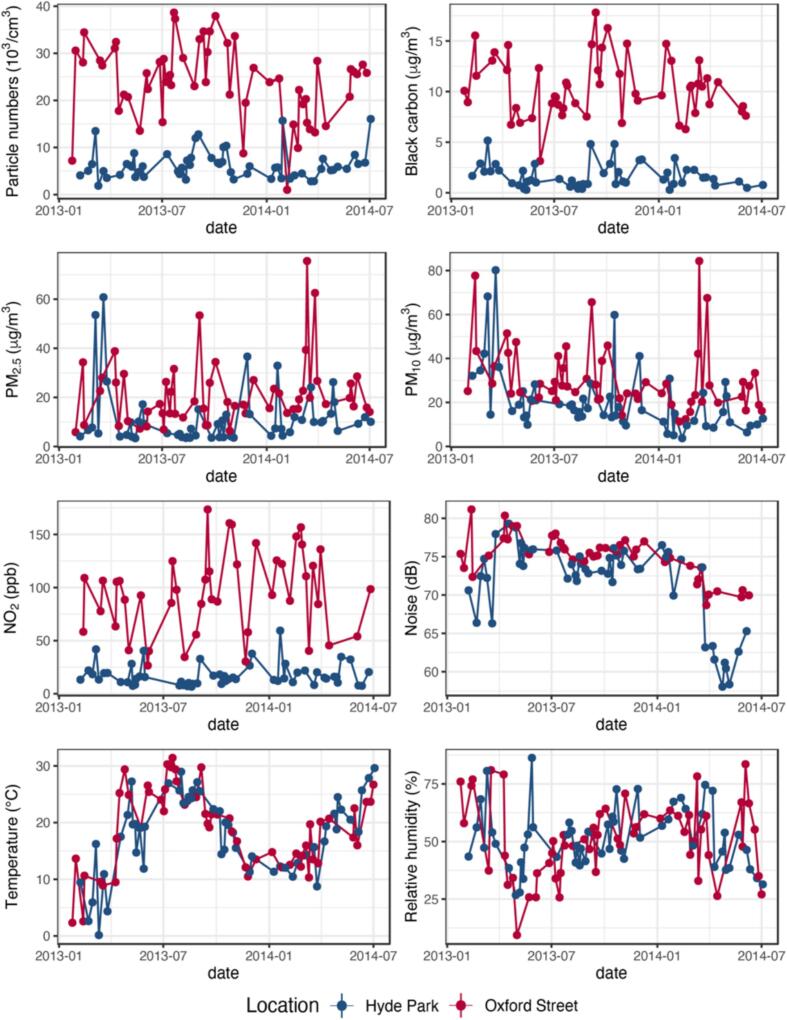
Exposure levels during the study days of various components of pollution during the 2-h walk on Oxford Street and in Hyde Park. PM_2.5_: particles <2.5 µm in diameter; PM_10_: particles <10 µm in diameter; NO_2_: nitrogen dioxide.

As depicted in Fig. 1, the 2-h average levels of PNC, black carbon, PM_2.5_, PM_10_, NO_2_, noise, temperature, and relative humidity at the two exposure sites were compared. The median concentrations of PNC, black carbon, and NO_2_ were 5.7 vs 25.4 × 10^3^/cm^3^, 1.3 vs 10.3 μg/m^3^, and 14.4 vs 95.5 ppb, respectively, which were significantly lower than those recorded on Oxford Street. Particulate matter and NO_2_ were correlated with each other, with Spearman correlation coefficients ranging from 0.44 to 0.84 (Supplemental Fig. S1). This disparity served as a clear indication of a typical traffic-polluted environment, predominantly characterized by diesel exhaust emissions. The overall exposure levels of PM_2.5_, PM_10_, and noise were modestly higher on Oxford Street. Meanwhile, no obvious differences were observed in terms of temperature or relative humidity.

### 3.2 Cardiac effects of walking between Oxford Street and Hyde Park

**Fig. 2:**
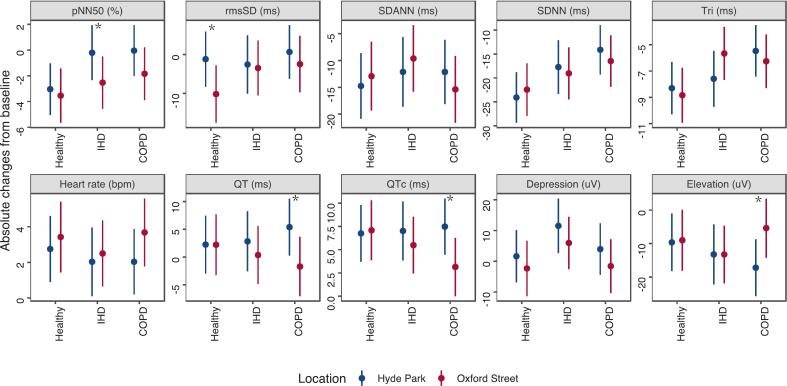
Absolute changes in cardiac responses over a 24-h period from the baseline measurement following a 2-h walk in Oxford Street or Hyde Park, stratified by disease status. *p < 0.05, comparing Oxford Street with Hyde Park. Data are shown as mean effect sizes and 95 % confidence intervals. No group-wise significance markers are shown in the figure as the between-group comparisons of pollution-related changes did not reach statistical significance (p ≥ 0.05).

We observed between-exposure site differences regarding the cardiac effects of the 2-h walk during the following 24 h in Fig. 2. Among the 3 disease groups, participants with COPD showed pronounced responses with higher heart rates, lower HRV, shortened QT intervals, and more depression and elevation of ST-segment after walking on Oxford Street in reference to walking in Hyde Park. There was a significant change in QTc from the baseline by 7.5 (95 %CI: 4.5 to 10.5) ms after the Hyde Park walk and a smaller change from the baseline by 3.1 (0.0 to 10.5) ms after the Oxford Street walk. We noted a reduction in ST elevation after the Oxford Street walk by −5.4 (−14.3 to 3.5) µV, with a significantly greater improvement compared with walking in Hyde Park by −17.2 (−25.7 to −8.7) µV. In participants with IHD, there were similar site differences in pNN50, with a greater decrease in Oxford Street by −2.5 % (−4.6 % to −0.5 %) compared to −0.2 % (−2.4 % to 1.9 %) in Hyde Park. We noted similar but non-significant trends in shortened QT intervals and more ST depression while on Oxford Street compared to Hyde Park. Healthy volunteers showed a significant decrease in rmsSD after the walk on Oxford Street by −10.2 (−17.6 to −2.8) ms, while there was no significant change after the Hyde Park walk. Between-group comparisons of pollution-related ECG changes are presented in Table 2. While these group-wise comparisons did not reach statistical significance, COPD participants exhibited numerically larger changes than healthy or IHD participants across most cardiac parameters (e.g., pNN50, QTc interval, and ST elevation), although the wide confidence intervals indicate substantial uncertainty.

**Table 2:** Between-group comparison of pollution-related ECG changes across disease groups.

Metrics	Group 1	Group 2	Difference	95 % CI	P value
Heart rate, bpm	COPD	Healthy	0.98	(−2.83, 4.79)	0.615
	COPD	IHD	1.18	(−2.60, 4.96)	0.540
	Healthy	IHD	0.21	(−3.63, 4.04)	0.916

pNN50, %	COPD	Healthy	−1.29	(−5.38, 2.79)	0.535
	COPD	IHD	0.53	(−3.59, 4.64)	0.801
	Healthy	IHD	1.82	(−2.33, 5.98)	0.390

rmsSD, ms	COPD	Healthy	5.86	(−8.51, 20.22)	0.424
	COPD	IHD	−2.22	(−16.64, 12.20)	0.763
	Healthy	IHD	−8.08	(−22.65, 6.49)	0.277

SDNN, ms	COPD	Healthy	−3.99	(−14.65, 6.68)	0.464
	COPD	IHD	−1.03	(−11.81, 9.75)	0.851
	Healthy	IHD	2.95	(−7.97, 13.88)	0.596

SDANN, ms	COPD	Healthy	−5.08	(−17.47, 7.31)	0.422
	COPD	IHD	−5.78	(−18.26, 6.71)	0.365
	Healthy	IHD	−0.69	(−13.35, 11.96)	0.914

Tri, ms	COPD	Healthy	−0.25	(−4.29, 3.80)	0.905
	COPD	IHD	−2.71	(−6.79, 1.37)	0.193
	Healthy	IHD	−2.46	(−6.58, 1.65)	0.241

QT, ms	COPD	Healthy	−7.06	(−17.67, 3.55)	0.192
	COPD	IHD	−4.62	(−15.19, 5.94)	0.391
	Healthy	IHD	2.44	(−8.24, 13.11)	0.654

QTc, ms	COPD	Healthy	−4.67	(−10.90, 1.56)	0.142
	COPD	IHD	−2.81	(−9.01, 3.40)	0.375
	Healthy	IHD	1.87	(−4.40, 8.13)	0.560

ST depression, μV	COPD	Healthy	−1.58	(−18.98, 15.83)	0.859
	COPD	IHD	0.04	(−17.30, 17.38)	0.997
	Healthy	IHD	1.61	(−15.91, 19.14)	0.857

ST elevation, μV	COPD	Healthy	11.18	(−6.38, 28.74)	0.212
	COPD	IHD	11.83	(−5.66, 29.33)	0.185
	Healthy	IHD	0.66	(−17.02, 18.33)	0.942

For each ECG metric, pollution-related change was calculated as the difference between post–pre values on Oxford Street minus that on Hyde Park. P-values refer to two-sided Z tests based on unpaired group differences; 95 % confidence intervals are shown in parentheses.

**Fig. 3:**
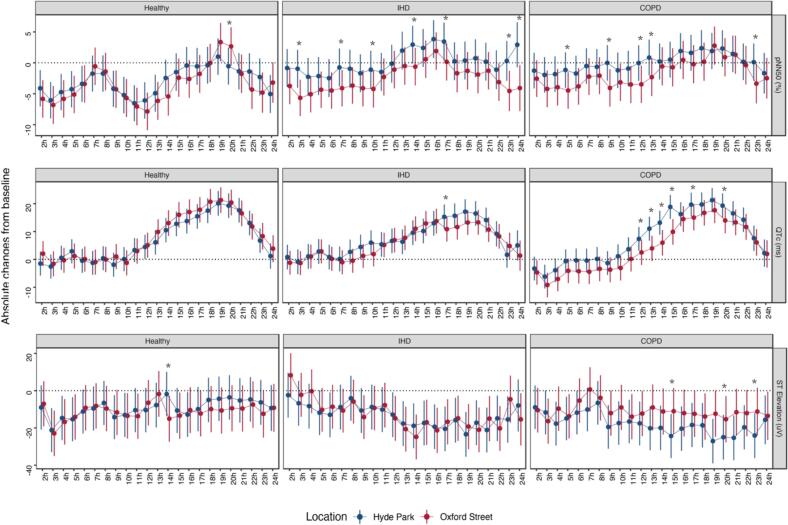
Hourly changes in heart rate, pNN50, and QTc from the baseline measurements during and following a 2-h walk in Oxford Street or Hyde Park, stratified by disease status. *p < 0.05, comparing Oxford Street with Hyde Park. Data were shown as mean effect sizes and 95 % confidence intervals. At 0 h, baseline measurements were taken before the start of the walk; at 2 h, the walk ended.

Given that pNN50, QTc intervals, and ST elevation are representative of HRV, ventricular repolarization, and myocardial injury, respectively, and have shown sensitivity and responsiveness during the 24 h from the baseline, an hourly-detailed analysis was conducted to further explore these effects (Fig. 3). We recorded significant site differences at several timepoints. For pNN50 compared to walking in Hype Park, we observed a 2.7 % reduction in hourly pNN50 (0.8 % to 7.0 %) among participants with IHD and a 1.8 % reduction (−0.8 % to 4.1 %) among participants with COPD while walking on Oxford Street. The site differences appeared soon after baseline and lasted for hours. Participants with COPD had more prominent site differences in QTc at later timepoints, including 12, 13, 14, 15, 17, and 20 h after the baseline by an average of 6.3 ms (4.6 ms to 9.0 ms; Fig. 3). For changes in ST elevation, we observed higher levels in participants with COPD walking on Oxford Street than on Hyde Park at 15, 20, and 23 h after the baseline. However, there were no such similar trends in participants with IHD and healthy volunteers.

After the 2-h walk, there were 29 episodes of ventricular ectopics observed with the Oxford Street session and 28 with the Hyde Park session. As for supraventricular ectopics, there were 21 cases associated with the Oxford Street walk and 27 with the Hyde Park walk. However, the logistic regression analysis did not reveal any significant differences in the occurrence of arrhythmic events between the two sites.

In terms of specific pollutant species (Supplemental Fig. S2), ultrafine PNC was associated with pNN50 reduction, QTc increase, and ST-segment elevation in patients with COPD and IHD. In addition, black carbon was associated with decreased pNN50 in COPD and IHD patients, shortened QTc, and elevated ST-segment in COPD patients. For example, an increase of 9.2 μg/m^3^ in black carbon was significantly associated with an increase of 10.3 (95 %CI: 1.2 to 19.5, p < 0.05) µV in ST elevation in COPD. In contrast, no significant associations were seen between any pollutant species and pNN50, QTc, and ST elevation in healthy participants.

### 3.3 Effect modification

**Fig. 4:**
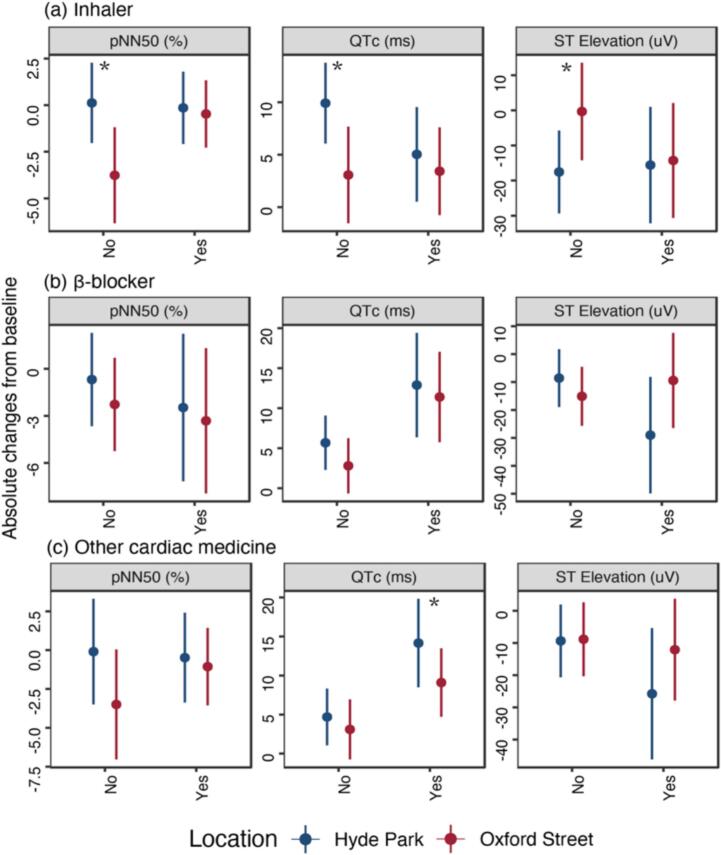
Effects modification by medication use on pNN50, QTc, and ST elevation. *p < 0.05, comparing Oxford Street with Hyde Park. We include COPD patients when examining the modifying effects of inhalers, and IHD patients when examining the modifying effects of β-blockers and other cardiac medicine. Data were shown as mean effect sizes and 95 % confidence intervals. Number of participants: COPD with inhaler: n = 28; COPD without inhaler: n = 10; IHD with β-blocker: n = 13; IHD without β-blocker: n = 26; IHD with other cardiac medicine: n = 15; IHD without other cardiac medicine: n = 24.

We examined whether medication use modified the cardiac effects induced by traffic pollution. For participants with COPD, medications included in our analysis were any of the inhaler drugs (short-acting beta-agonist, long-acting β-agonist, long-acting muscarinic antagonist, corticosteroid, and combined corticosteroid and long-acting beta agonist). For participants with IHD, medications considered in our analysis were β-blockers and/or other cardiac medicines (Fig. 4). Participants with COPD who did not use inhaler drugs had significantly lower pNN50 and shorter QTc and higher ST elevation after walking on Oxford Street compared to the Hyde Park walk. By contrast, participants with COPD who used inhalers did not show significant differences comparing the two sites. Participants with IHD who did not use β-blockers and other cardiac medicine had lower pNN50 after walking on Oxford Street than in Hyde Park, while IHD participants without medication use did not have worse changes in QTc and ST elevation. Although not statistically significant, post-hoc comparisons showed more favorable ECG responses among inhaler users than non-users in COPD participants (Supplementary Table S2).

### 3.4 Urinary NO_2_-cLA

**Fig. 5:**
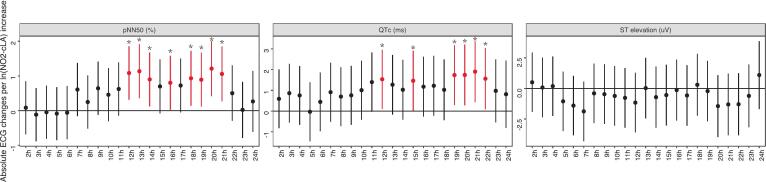
Associations between NO_2_-cLA and ECG changes in participants with COPD. *p < 0.05. Data were shown as mean effect sizes and 95 % confidence intervals.

COPD participants exhibited the highest baseline level of urinary NO_2_-cLA compared to healthy volunteers and IHD participants (Table 1). However, NO_2_-cLA levels were significantly lower in COPD participants after walking on Oxford Street compared to walking in Hyde Park (median values 0.0189 vs 0.0323 μg/g creatinine, mixed-effects model *p* = 0.03; Supplemental Table S3), whereas no significant differences were observed between these conditions in healthy volunteers and IHD participants. Furthermore, in COPD participants, NO_2_-cLA measured 24 h after exposure was positively associated with increases in pNN50 and QTc and a reduction in ST elevation (Fig. 5). In particular, between 12 and 22 h post-exposure, the association between NO_2_-cLA and increases in pNN50 and QTc was most pronounced (*p* < 0.05), with a one-unit increase in log-transformed NO_2_-cLA concentration corresponding to approximately a 1 % increase in pNN50 and a 1.5 ms increase in QTc.

## 4 Discussion

In this randomized crossover study, we showed that a 2-h walk in a busy traffic environment led to adverse cardiac activities, including an accelerated heart rate, reduced HRV, shortened QT intervals, and increased ST-segments on ECGs, although we did not find a significant increase in arrhythmic events. Among the measured pollutants, the strongest associations with electrocardiographic alterations were seen for ultrafine particles, black carbon, and NO_2_, consistent with their role as markers of diesel exhaust and traffic-related air pollution. While some ECG changes were larger in COPD patients, between-group differences were not statistically significant, limiting conclusions about differential responsiveness. The observed differences in COPD patients may be associated with altered levels of cardioprotective molecule NO_2_-cLA following pollution exposure, as one possible mechanistic explanation. We found that COPD participants using inhalers containing β-agonists, anticholinergics, and corticosteroids exhibited less pronounced ECG changes, including smaller reductions in HRV and QTc intervals, following exposure to traffic-related pollution compared to those not on these medications.

Our quasi-experimental design contributes to the growing body of research investigating the cardiovascular effects of air pollution in real-world settings. Unlike controlled human exposure studies, such as diesel exhaust chamber experiments that offer rigorous control of exposure conditions and participant activity levels (Cosselman et al., 2012, Vieira et al., 2016, Sack et al., 2016), our study captures physiological responses under naturalistic urban exposure scenarios. This complementary approach allows for assessment of subclinical and molecular cardiovascular changes during common daily activities such as roadside commuting and outdoor walking, thereby improving public health relevance. In addition to its real-world design, our study uniquely contributes by directly comparing individuals with COPD, IHD, and healthy controls within the same experimental framework, and by exploring potential protective mechanisms, including the use of inhaled or cardiac medications and the modulation of NO_2_-cLA, a cardioprotective lipid mediator.

Our findings extend current understanding of differential cardiac responses to traffic pollution in this framework of experimental studies. Elevated heart rate and reduced HRV, both established indicators of cardiovascular risk, have been extensively studied in environmental epidemiological research (Jouven et al., 2005, La Rovere et al., 2003, Fox et al., 2007). These indicators suggest an autonomic imbalance; which is a critical mechanism linking air pollution and noise exposure to cardiovascular effects (Munzel et al., 2017, Rajagopalan et al., 2018). Our study demonstrated that participants with COPD and IHD experienced greater increments in heart rate and decrements in HRV after walking on Oxford Street compared to walking in Hyde Park. Furthermore, the effects of air pollution on cardiac repolarization parameters have not been as extensively studied as HRV. Early research reported associations between short- and long-term exposure to air pollutants like PM_2.5_ and an extended QT interval (Liao et al., 2010, Mordukhovich et al., 2016), while a few observational studies recently reported an inverse relationship between air pollutants and the QT interval (Bind et al., 2016, Gondalia et al., 2021). We provide evidence that COPD participants showed reductions in QTc intervals following exposure, reflecting subclinical electrophysiological alterations in response to environmental exposure. Although prolonged QT intervals are widely recognized as a marker of arrhythmia risk, emerging evidence also suggests that abnormally shortened QT intervals can reflect accelerated myocardial repolarization and are associated with an increased risk of ventricular fibrillation and sudden cardiac death (Tanti et al., 2022, Zhang et al., 2011). Furthermore, we observed small, subclinical increases in ST-segment elevation following traffic pollution exposure, particularly in COPD patients. While epidemiological studies have reported associations between PM exposure and increased risk of ST-elevation myocardial infarction, these studies refer to clinical events (Pope et al., 2015, Pan et al., 2019, Sahlen et al., 2019). These minor ST-segment elevations may reflect subclinical electrophysiological perturbations, though their clinical significance remains to be determined in future studies focusing on arrhythmic risk.

Previous research suggested that older adults and those with cardiac or metabolic diseases as susceptible populations to air pollution-related cardiovascular effects (Newman et al., 2020). Our study contributes to this body of evidence by suggesting that COPD participants may be more susceptible, as they showed numerically greater electrophysiological changes compared to those with IHD, though these differences were not statistically significant. These preliminary observations suggest COPD patients may potentially represent a susceptible population, although larger studies are needed to confirm differences. Several factors may contribute to this heightened susceptibility. Firstly, among the three groups in this study, COPD patients had the worst baseline cardiac electrophysiological profiles, including lower HRV (e.g., pNN50, rmsSD, SDNN) and a trend toward longer QTc intervals, suggesting reduced cardiac resilience and physiological reserve (Bind et al., 2016, Geller and Zenick, 2005). Second, in COPD patients, the presence of airflow obstruction may increase the interception of larger particles in the upper respiratory tract, but enhance the retention of fine particles in peripheral airways (Luo et al., 2007, Löndahl et al., 2012). Notably, ultrafine particle concentrations were profoundly higher on Oxford Street than in Hyde Park, among other measured pollutants, implying the importance of ultrafine particle exposure in close proximity to tailpipe emissions during a road-side walk. Ultrafine particles are thought to have greater pro-oxidative potential, deeper pulmonary penetration, and even systemic translocation (Miller and Newby, 2020, Ohlwein et al., 2019), factors that make them especially concerning for individuals with structurally compromised airways like COPD patients. Importantly, cardiovascular complications are a major cause of morbidity and mortality in COPD patients, surpassing deaths due to respiratory failure (Sin and Man, 2005, Chen et al., 2015). Our findings underscore the need to consider COPD not only as a respiratory condition but also as a cardiovascularly vulnerable population in the context of traffic-related air pollution exposure. This has important implications for risk stratification and the development of targeted prevention strategies.

After developing chronic cardiopulmonary diseases, individuals often take regular medication for years to manage their condition. These medications may alter their responses to air pollution exposure. We found that participants with COPD using inhaler drugs did not exhibit significant cardiac differences after exposure to traffic pollution compared to walking in green spaces, which raises the possibility that this inhaler therapy may protect against the cardiac effects of traffic pollution exposure. β-agonists, anticholinergics, and inhaled corticosteroids are commonly used in the treatment of obstructive airway disease such as asthma and COPD. Asthmatic patients use their β-agonist inhalers more often after air pollution exposure (Williams et al., 2019, Gent et al., 2009). The β-agonist inhaler could improve patients' lung function, and the improved oxygenation status may help maintain normal electrophysiological function of the heart. Inhaled corticosteroids exert anti-inflammatory effects, thereby alleviating the inflammatory initiation phase of adverse cardiovascular effects caused by air pollution (Brook et al., 2010). Yet there are concerns about the increased cardiovascular risks provoked by β-agonists and anticholinergics (Gershon et al., 2013, Wood-Baker et al., 2010). To the contrary, we found no difference between IHD participants taking or not taking β-blockers in the QT interval and ST-segment elevation after traffic exposure. There are conflicting reports regarding the effect of β-blockers in their potential protective actions against air pollution exposure (Pekkanen et al., 2002, Long et al., 2008, Wheeler et al., 2006). In our study, IHD patients using β-blockers exhibited lower baseline HRV (e.g., SDNN and SDANN) and more negative ST-segment depression compared to non-users (Supplementary Table S4). We cannot conclude whether this lack of difference between those taking and not taking β-blockers was due to the ineffectiveness of β-blockers or simply reflected the inherent vulnerability of patients with already compromised health. We did not incorporate time-specific medication use into the hour-by-hour ECG analysis due to incomplete reporting of administration timing, which limits our ability to evaluate acute pharmacodynamic interactions with exposure.

NO_2_-cLA is a nitro-conjugated linoleic acid derivative that has recently gained attention due to its anti-inflammatory and cardiovascular protective properties (Villacorta et al., 2016). Cardiac injuries lead to increased endogenous biosynthesis of NO_2_-cLA which exhibits cardiovascular protective effects, such as improving vascular function and reducing arrhythmias and myocardial infarction (Mollenhauer et al., 2018). Exogenous NO_2_-cLA intake has also been shown to improve cardiac function (Rudolph et al., 2010). We therefore investigated the potential role of NO_2_-cLA on the electrocardiographic effects induced by air pollution. In our analysis, exposure to traffic pollution led to significant decreases in urinary NO_2_-cLA levels among COPD patients, which was associated with adverse ECG changes such as HRV and QTc. Because air pollution did not cause NO_2_-cLA changes in healthy or IHD participants, NO_2_-cLA may explain, at least partially, why COPD patients exhibited more pronounced ECG changes following traffic exposure.

Although the participants experienced episodes of tachyarrhythmias and bradyarrhythmias, we did not observe a significant difference in the incidence of arrhythmic events between walking on the traffic-polluted street and walking on the traffic-free park. The association between air pollution and ventricular arrhythmias remains vague (Newby et al., 2015), partially due to the duration of exposure or the time window of effects. While controlled-exposure studies have not consistently shown an association between short-term air pollution exposure and arrhythmias, (Rich et al., 2005, Ljungman et al., 2008, Link et al., 2013, Langrish et al., 2014) an association between PM_2.5_ exposure and increased onset of arrhythmias has been reported in large recent cohort series (Kim et al., 2019, Shin et al., 2019, Zhang et al., 2020).

Our study has several limitations. First, the measured pollutants may not sufficiently capture the complexity of traffic-related exposures, as perceptions or stress related to traffic pollution can also contribute to cardiovascular burden. Moreover, pollution levels in this study reflect traffic conditions in London during 2012–2014, when diesel vehicles were more common and ultrafine particles likely dominated, which may differ from other traffic emission profiles. Second, occasional signal artifacts, such as motion-related distortions or electrode contact issues, were present in the Holter monitor recordings. However, all ECG data were reviewed by the cardiologist (Dr. Sabine Ernst), blinded to exposure conditions, to ensure data integrity. Third, medication use was not standardized, reflecting real-world variability, but future research should investigate how different treatments may mitigate pollution-induced cardiovascular effects. Fourth, the study was not powered to detect effect modification across disease groups. While COPD participants showed some numerically larger ECG responses, the between-group comparisons did not reach statistical significance, and confidence intervals were wide and overlapping. As such, observed group differences should be interpreted as exploratory and hypothesis-generating rather than conclusive. Fifth, the presence of asymptomatic IHD could not be ruled out in participants with COPD. However, undiagnosed cardiovascular comorbidities are common in COPD patients and reflect the intrinsic cardiopulmonary vulnerability of this population (Chen et al., 2015). This overlap aligns with the real-world clinical heterogeneity that our study aimed to capture. Finally, our study did not control for rest or varying intensities of exercise, focusing instead on the comparative impact of pollution across two sites (the street versus the park). It is important to note that the electrophysiological changes observed in this study reflect acute responses to short-term exposure and were measured in a controlled, time-limited context. While these findings suggest transient perturbations in cardiac autonomic and repolarization dynamics, their long-term significance is not yet clear. Acute physiological responses, such as those seen during exercise, may be adaptive in certain contexts. Further longitudinal research is needed to determine whether repeated exposure to traffic-related pollution and associated subclinical electrical changes contribute to long-term cardiovascular risk.

This randomized crossover study offers novel insights into the acute cardiac electrophysiologic responses to traffic-related air pollution in participants with COPD, IHD, and healthy controls. COPD participants showed larger changes than healthy participants in some cardiac parameters, although between-group differences were not statistically significant. These preliminary observations warrant investigation in larger, adequately powered studies. The heightened responses in COPD participants were also reflected in greater depletion of NO_2_-cLA, a cardioprotective molecule, suggesting a compromised biological defense against pollution-induced stress. It underscores the importance of considering disease-specific susceptibility in environmental health research. Future studies with larger cohorts and extended follow-up are warranted to further elucidate the pathways of susceptibility and evaluate protective strategies for at-risk populations.

## CRediT authorship contribution statement

**Xin Meng:** Writing – review & editing, Writing – original draft, Formal analysis. **Yan Lin:** Writing – review & editing, Investigation. **Jicheng Gong:** Writing – review & editing, Supervision, Methodology, Conceptualization. **Peter Collins:** Methodology, Investigation, Data curation, Conceptualization. **Sabine Ernst:** Writing – review & editing, Methodology, Data curation, Conceptualization. **Wu Chen:** Writing – review & editing. **Meilin Yan:** Writing – review & editing. **Junfeng Jim Zhang:** Writing – review & editing, Supervision, Methodology, Funding acquisition, Conceptualization. **Kian Fan Chung:** Writing – review & editing, Supervision, Methodology, Funding acquisition, Conceptualization.

## Declaration of competing interest

The authors declare that they have no known competing financial interests or personal relationships that could have appeared to influence the work reported in this paper.

## Data Availability

Data will be made available on request.
