# Supplementary material for: Cardiac electrophysiological responses to traffic pollution in adults with or without chronic cardiopulmonary diseases
Source: Environ Int. 2025 Sep;203:109764. doi: 10.1016/j.envint.2025.109764 (PMC12450113; doi:10.1016/j.envint.2025.109764)
Supplement: Supplementary Data 1 [file mmc1.docx]

**Supplementary Information**

**Susceptibility to Cardiac Effects of Traffic Pollution in Adults with or without Chronic Cardiopulmonary Diseases**

Xin Meng, PhD, ^a^ Yan Lin, PhD, ^b^ Jicheng Gong, PhD, ^a^ Peter Collins, MD, ^c^ Sabine Ernst, MD, ^c^ Wu Chen, PhD, ^a, d^ Meilin Yan, PhD, ^e^ Junfeng (Jim) Zhang, PhD, ^b^ Kian Fan Chung, MD^c^

^a^ SKL-ESPC & SEPKL-AERM, College of Environmental Sciences and Engineering, and Center for Environment and Health, Peking University, Beijing, China

^b^ Nicholas School of the Environment & Duke Global Health Institute, Duke University, Durham, NC, USA

^c^ National Heart & Lung Institute, Imperial College London & Royal Brompton & Harefield Hospital, London, UK

^d^ Department of Population and Public Health Sciences, Keck School of Medicine, University of Southern California, Los Angeles, California, USA

^e^ Department of Environmental Science and Engineering, School of Light Industry Science and Engineering, Beijing Technology and Business University, Beijing, China

**Address for correspondence:** Corresponding author - Jicheng Gong, Associate Professor, Peking University, [jicheng.gong@pku.edu.cn](mailto:jicheng.gong@pku.edu.cn); Senior author - Kian Fan Chung, Professor, Imperial College London, [f.chung@imperial.ac.uk](mailto:f.chung@imperial.ac.uk)

**Table S1. Summary of baseline ECG levels comparing walking in Hyde Park and Oxford Street.**

|  | Hyde Park | Oxford Street | Overall |
| --- | --- | --- | --- |
| Heart rate, bpm |  |  |  |
| Mean (SD) | 71.1 (11.2) | 70.8 (11.6) | 70.9 (11.4) |
| Median [Min, Max] | 70.0 [48.0, 100] | 69.5 [43.0, 107] | 70.0 [43.0, 107] |
| pNN50, % |  |  |  |
| Mean (SD) | 8.28 (12.4) | 9.18 (13.2) | 8.72 (12.8) |
| Median [Min, Max] | 4.00 [0, 85.0] | 4.00 [0, 72.0] | 4.00 [0, 85.0] |
| rmsSD, ms |  |  |  |
| Mean (SD) | 41.8 (58.3) | 43.7 (49.3) | 42.7 (53.9) |
| Median [Min, Max] | 28.0 [2.00, 469] | 27.0 [2.00, 310] | 28.0 [2.00, 469] |
| SDNN, ms |  |  |  |
| Mean (SD) | 70.0 (26.9) | 68.2 (23.0) | 69.1 (25.0) |
| Median [Min, Max] | 67.0 [25.0, 228] | 65.5 [3.00, 126] | 66.0 [3.00, 228] |
| SDANN, ms |  |  |  |
| Mean (SD) | 63.8 (22.4) | 63.9 (23.0) | 63.8 (22.6) |
| Median [Min, Max] | 62.0 [24.0, 135] | 60.5 [22.0, 134] | 61.0 [22.0, 135] |
| Tri, ms |  |  |  |
| Mean (SD) | 22.4 (7.66) | 22.2 (8.04) | 22.3 (7.83) |
| Median [Min, Max] | 22.0 [8.00, 43.0] | 21.0 [8.00, 51.0] | 21.0 [8.00, 51.0] |
| QT, ms |  |  |  |
| Mean (SD) | 397 (28.4) | 399 (29.8) | 398 (29.1) |
| Median [Min, Max] | 398 [326, 466] | 398 [324, 473] | 398 [324, 473] |
| QTc, ms |  |  |  |
| Mean (SD) | 414 (18.9) | 416 (19.9) | 415 (19.4) |
| Median [Min, Max] | 414 [365, 501] | 413 [377, 484] | 414 [365, 501] |
| ST depression, μV |  |  |  |
| Mean (SD) | -55.4 (46.5) | -51.6 (47.1) | -53.6 (46.7) |
| Median [Min, Max] | -46.0 [-352, -10.0] | -39.0 [-440, -14.0] | -42.0 [-440, -10.0] |
| ST elevation, μV |  |  |  |
| Mean (SD) | 132 (76.8) | 128 (79.8) | 130 (78.1) |
| Median [Min, Max] | 113 [33.0, 448] | 110 [28.0, 606] | 113 [28.0, 606] |

**Table S2. Comparison of exposure-related ECG changes between medication users and non-users in COPD and IHD participants**

| Medicine | Metrics | Difference | 95% CI | P value |
| --- | --- | --- | --- | --- |
| Inhalers | Heart rate (bpm) | -0.82 | (-6.42, 4.79) | 0.775 |
|  | pNN50 (%) | -3.56 | (-7.83, 0.72) | 0.103 |
|  | rmsSD (ms) | -7.31 | (-17.69, 3.07) | 0.167 |
|  | SDNN (ms) | -6.57 | (-20.91, 7.76) | 0.369 |
|  | SDANN (ms) | -7.25 | (-22.86, 8.36) | 0.363 |
|  | Tri (ms) | -0.96 | (-6.24, 4.33) | 0.722 |
|  | QT (ms) | -4.14 | (-18.99, 10.72) | 0.585 |
|  | QTc (ms) | -5.24 | (-13.82, 3.35) | 0.232 |
|  | ST Depression (uV) | -5.04 | (-27.28, 17.19) | 0.656 |
|  | ST Elevation (uV) | 15.92 | (-13.65, 45.48) | 0.291 |
| β-blocker | Heart rate (bpm) | -1.74 | (-9.19, 5.71) | 0.647 |
|  | pNN50 (%) | -0.74 | (-8.56, 7.08) | 0.852 |
|  | rmsSD (ms) | 2.23 | (-24.35, 28.81) | 0.869 |
|  | SDNN (ms) | -5.28 | (-19.46, 8.90) | 0.465 |
|  | SDANN (ms) | 3.75 | (-14.25, 21.74) | 0.683 |
|  | Tri (ms) | -1.33 | (-7.52, 4.86) | 0.673 |
|  | QT (ms) | 2.62 | (-16.58, 21.82) | 0.789 |
|  | QTc (ms) | -1.39 | (-11.27, 8.49) | 0.783 |
|  | ST Depression (uV) | -2.64 | (-33.08, 27.80) | 0.865 |
|  | ST Elevation (uV) | -26.1 | (-56.82, 4.63) | 0.096 |
| Other cardiac medicine | Heart rate (bpm) | 0.49 | (-5.61, 6.59) | 0.876 |
|  | pNN50 (%) | -2.82 | (-9.03, 3.39) | 0.374 |
|  | rmsSD (ms) | -0.12 | (-23.35, 23.11) | 0.992 |
|  | SDNN (ms) | -4.35 | (-18.60, 9.90) | 0.550 |
|  | SDANN (ms) | -17.59 | (-38.55, 3.38) | 0.100 |
|  | Tri (ms) | -0.95 | (-6.86, 4.95) | 0.752 |
|  | QT (ms) | 2.19 | (-13.73, 18.11) | 0.787 |
|  | QTc (ms) | 3.47 | (-5.43, 12.37) | 0.445 |
|  | ST Depression (uV) | 24.14 | (-25.74, 74.02) | 0.343 |
|  | ST Elevation (uV) | -13.18 | (-43.56, 17.19) | 0.395 |

For each ECG metric, the pollution-related change was defined as the difference in pre–post values between Oxford Street and Hyde Park exposures. For each medication category (inhalers in COPD; β-blockers and other cardiac medications in IHD), we compared the exposure-related ECG changes between medication users and non-users. P-values refer to two-sided Z tests based on unpaired group differences; 95% confidence intervals are shown in parentheses.

**Table S3. Urinary NO_2_-cLA concentrations at baseline and 24 hours after exposure comparing walking in Hyde Park and Oxford Street**

|  | Hyde Park | | |  | Oxford Street | | | *p* value^b^ |
| --- | --- | --- | --- | --- | --- | --- | --- | --- |
|  | Baseline | 24h after | *p* value^a^ |  | Baseline | 24h after | *p* value^a^ |  |
| Healthy | 0.0182 (0.0377) | 0.0150 (0.0665) | 0.555 |  | 0.00664 (0.0647) | 0.0214 (0.140) | 0.014 | 0.327 |
| IHD | 0.00954 (0.0571) | 0.0297 (0.0948) | 0.637 |  | 0.0145 (0.0647) | 0.0115 (0.0888) | 0.956 | 0.659 |
| COPD | 0.0202 (0.205) | 0.0323 (0.204) | 0.352 |  | 0.0245 (0.154) | 0.0189 (0.0758) | 0.044 | 0.031 |

NO_2_-cLA concentrations (unit: μg/g creatinine) were shown as median (IQR). Differences were compared using mixed-effect models.

^a^ Comparison of NO_2_-cLA concentrations between baseline and 24 hours after walk.

^b^ Comparison of NO_2_-cLA concentrations measured 24 hours after walking on Oxford Street versus after walking in Hyde Park.

**Table S4. Summary of baseline ECG levels comparing IHD participants using β-blockers.**

|  | Without β-blockers | With β-blockers |
| --- | --- | --- |
| Heart rate, bpm |  |  |
| Mean (SD) | 64.3 (9.84) | 67.8 (9.31) |
| Median [Min, Max] | 62.0 [43.0, 99.0] | 66.5 [50.0, 84.0] |
| pNN50, % |  |  |
| Mean (SD) | 8.94 (13.3) | 5.67 (8.20) |
| Median [Min, Max] | 4.00 [0, 72.0] | 2.50 [0, 30.0] |
| rmsSD, ms |  |  |
| Mean (SD) | 45.3 (52.4) | 30.7 (29.9) |
| Median [Min, Max] | 26.0 [12.0, 327] | 25.0 [10.0, 143] |
| SDNN, ms |  |  |
| Mean (SD) | 73.4 (27.6) | 66.9 (20.0) |
| Median [Min, Max] | 71.0 [37.0, 228] | 67.0 [35.0, 100] |
| SDANN, ms |  |  |
| Mean (SD) | 69.1 (21.9) | 56.7 (20.0) |
| Median [Min, Max] | 66.0 [36.0, 135] | 52.5 [28.0, 98.0] |
| Tri, ms |  |  |
| Mean (SD) | 23.2 (6.21) | 21.0 (6.57) |
| Median [Min, Max] | 21.5 [12.0, 40.0] | 21.0 [10.0, 34.0] |
| QT, ms |  |  |
| Mean (SD) | 416 (27.5) | 408 (25.9) |
| Median [Min, Max] | 418 [339, 473] | 410 [360, 464] |
| QTc, ms |  |  |
| Mean (SD) | 420 (24.3) | 421 (25.5) |
| Median [Min, Max] | 418 [365, 501] | 419 [377, 484] |
| ST depression, μV |  |  |
| Mean (SD) | -68.7 (69.6) | -51.8 (38.2) |
| Median [Min, Max] | -49.0 [-440, -11.0] | -34.5 [-141, -13.0] |
| ST elevation, μV |  |  |
| Mean (SD) | 146 (94.9) | 145 (90.7) |
| Median [Min, Max] | 116 [48.0, 606] | 145 [44.0, 420] |


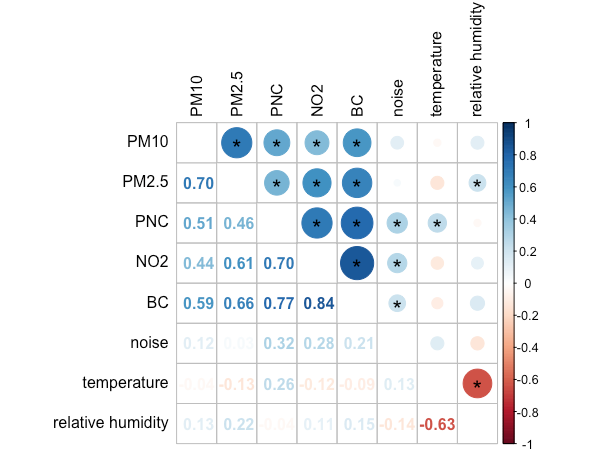


PNC denotes particle number concentration; BC, black carbon.

* It represents the statistical significance at the level of 0.05.

**Figure S1. Spearman correlation of measured air pollutants, noise, and meteorological parameters on the walk days.**

**Figure S2. Estimated means and 95% CIs for changes in pNN50, QTc, and ST elevation with one IQR increase in pollutants**

*p<0.05. Data were shown as mean effect sizes and 95% confidence intervals.

PNC=particle number concentration (per 19854/cm³). BC=black carbon (per 9.2 μg/m³). PM_2.5_=particles <2.5 µm in diameter (per 14.94 μg/m³). PM_10_=particles <10 µm in diameter (per 14.47 μg/m³). NO_2_=nitrogen dioxide (per 64.9 ppb). Noise per 3.97 dB.
